# Supplementary material for: Drop it all: extraction-free detection of targeted marine species through optimized direct droplet digital PCR
Source: PeerJ. 2024 Feb 23;12:e16969. doi: 10.7717/peerj.16969 (PMC10896080; doi:10.7717/peerj.16969)
Supplement: Supplemental Information 3 [file peerj-12-16969-s003.docx]

**Supplemental Information for:**

Drop it all: Extraction-free detection of targeted marine species through optimized direct-digital droplet PCR

**Supplementary Information File 1: Figures and Tables**

**
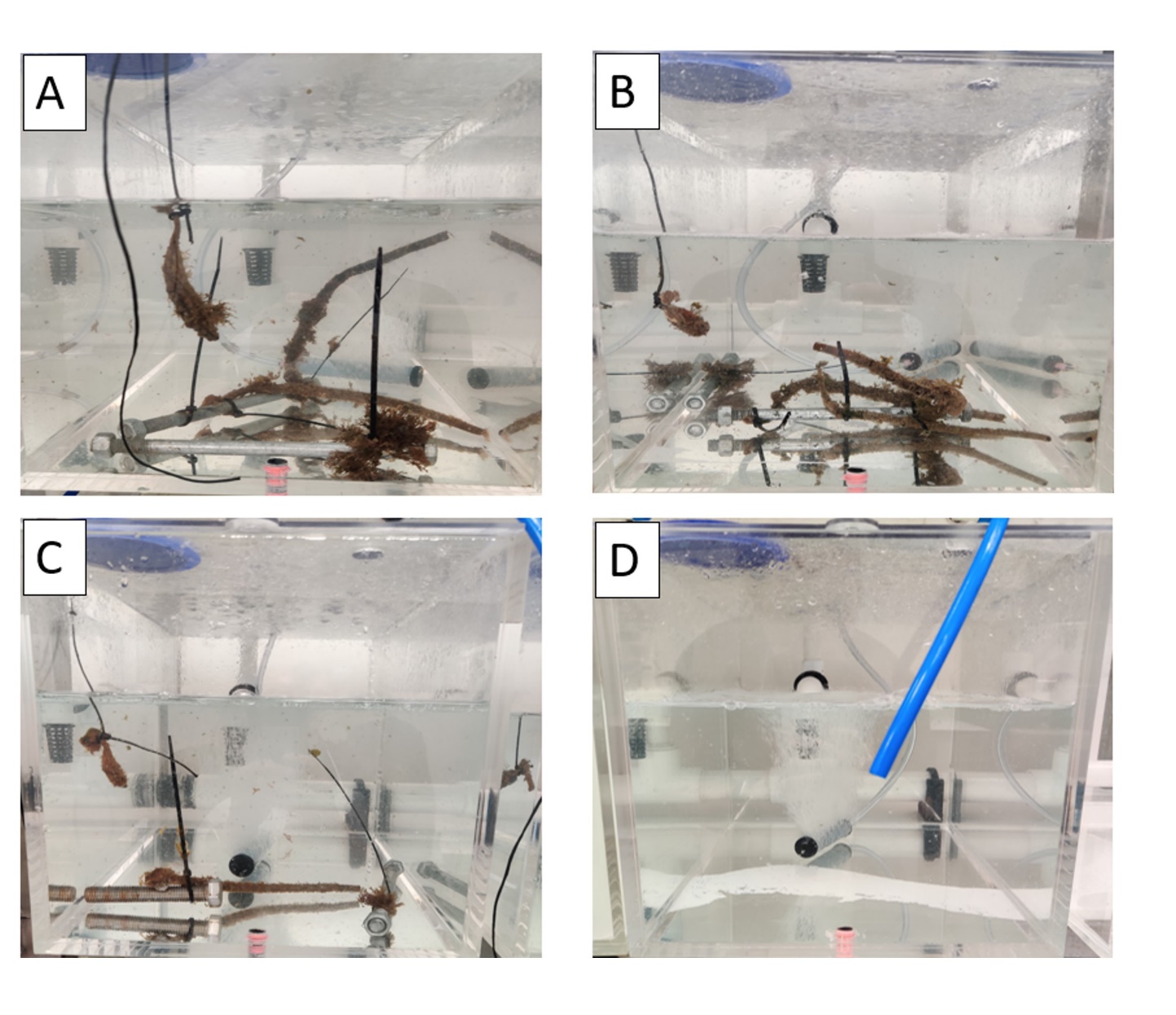
**

**Figure S1.** Photos taken of *Sabella spallanzanii*, *Bugula neritina* and *Styela clava* during the aquarium experiments. Photo of tanks taken at the start of the experiment time point (0 hours); (**A**) Tank 1, (**B**) Tank 2, (**C**) Tank 3, and (**D**) Tank 4 as a negative control.


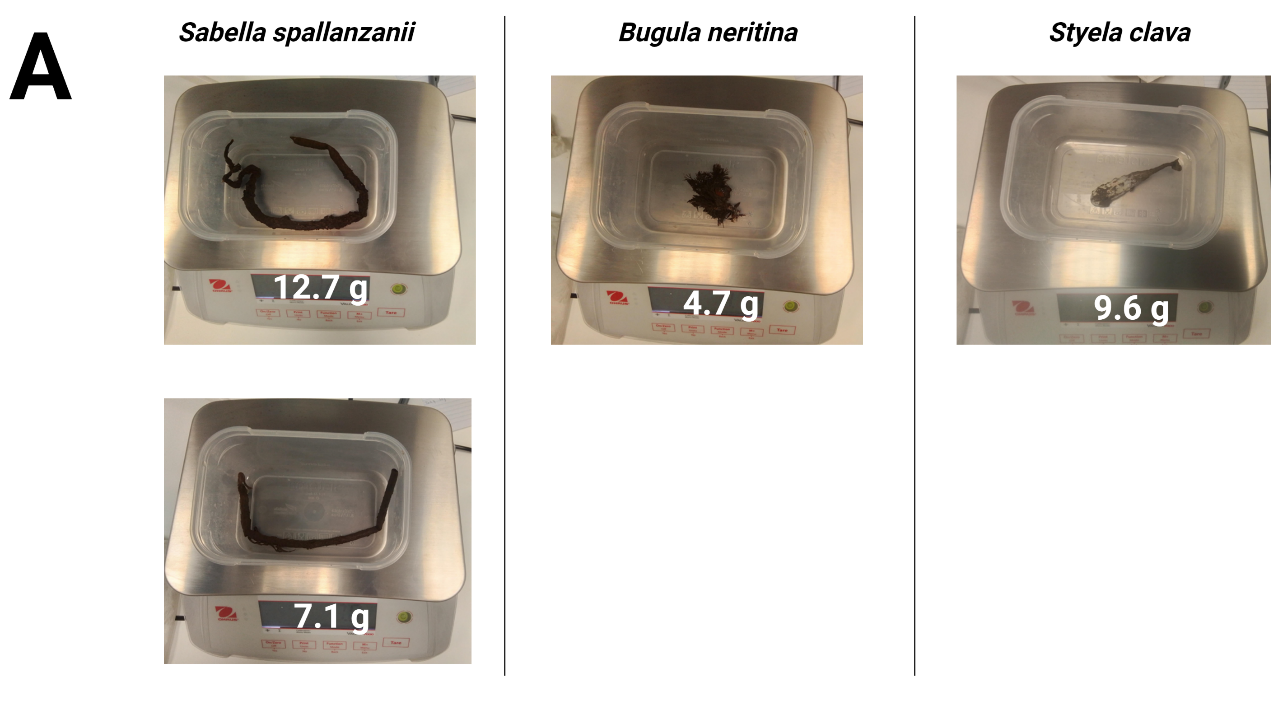


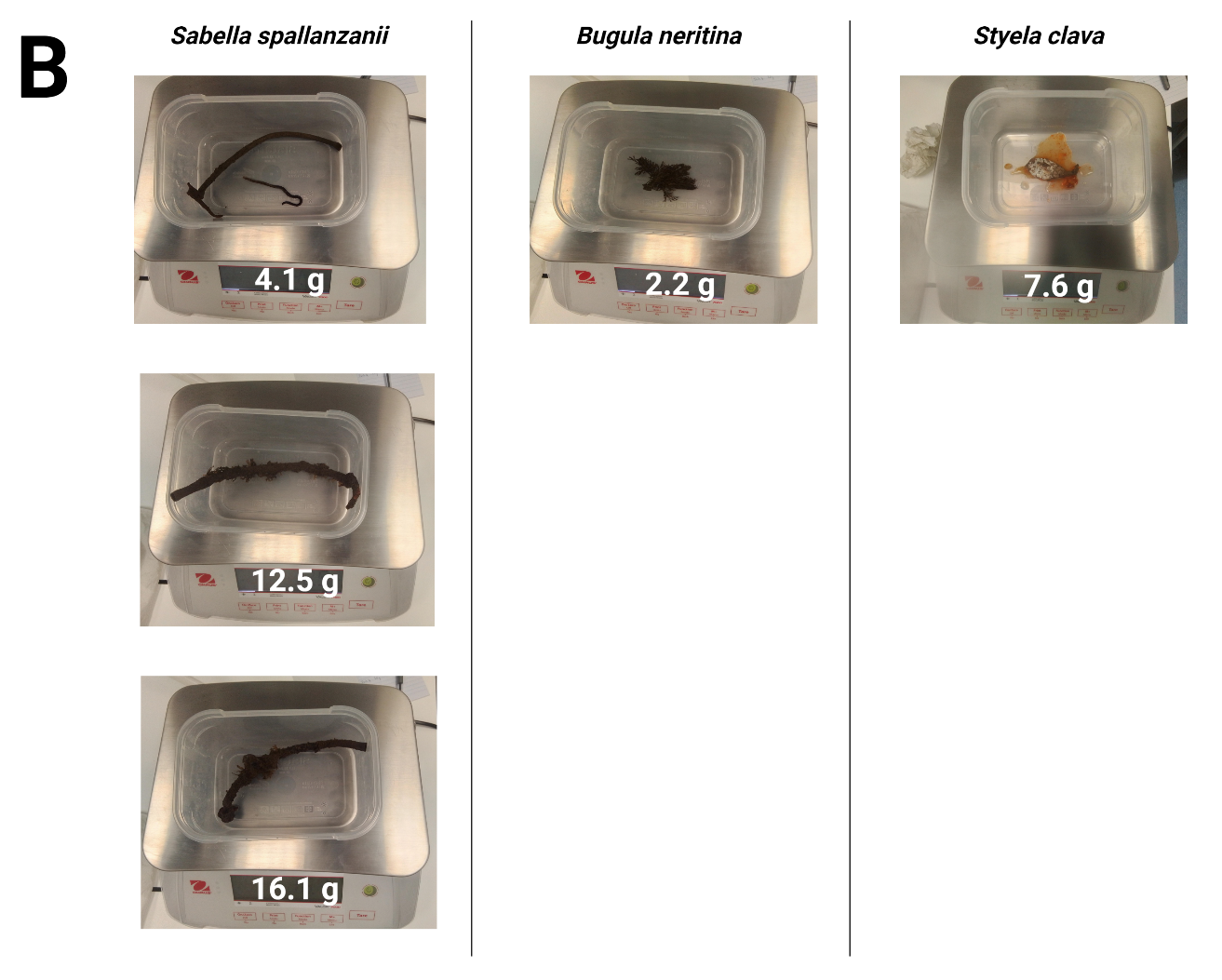


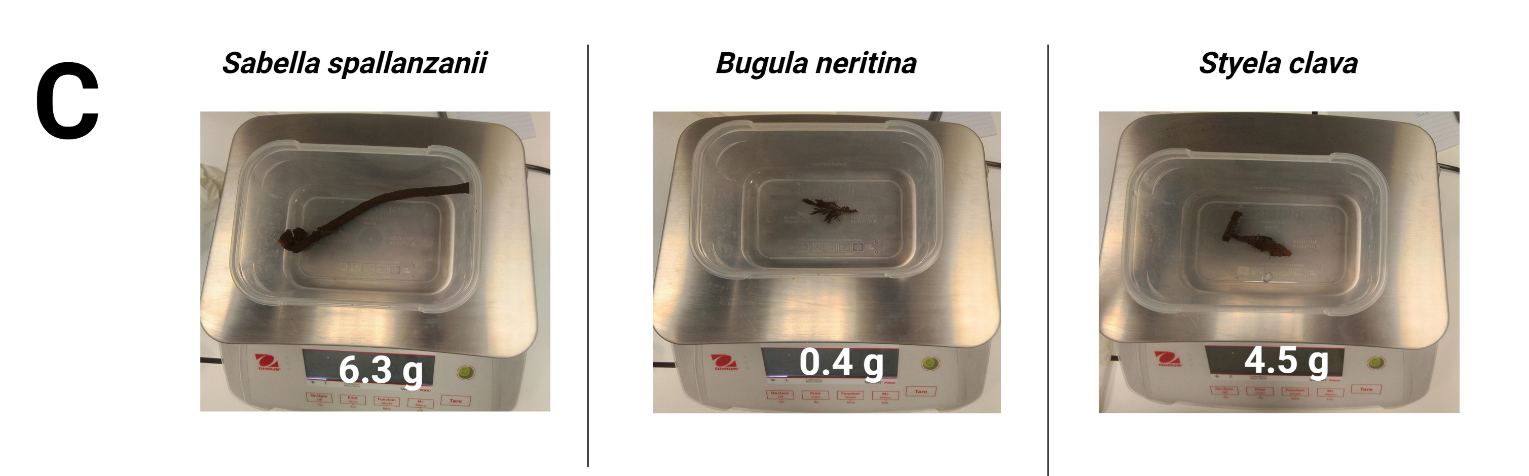
**Figure S2**. Photos taken of *Sabella spallanzanii*, *Bugula neritina* and *Styela clava* after being removed from Tank 1 (**A**), Tank 2 (**B**), and Tank 3 (**C**). After being removed from the aquarium, organisms were weighed; the weight of the organisms is noted in the figure at the bottom.

**
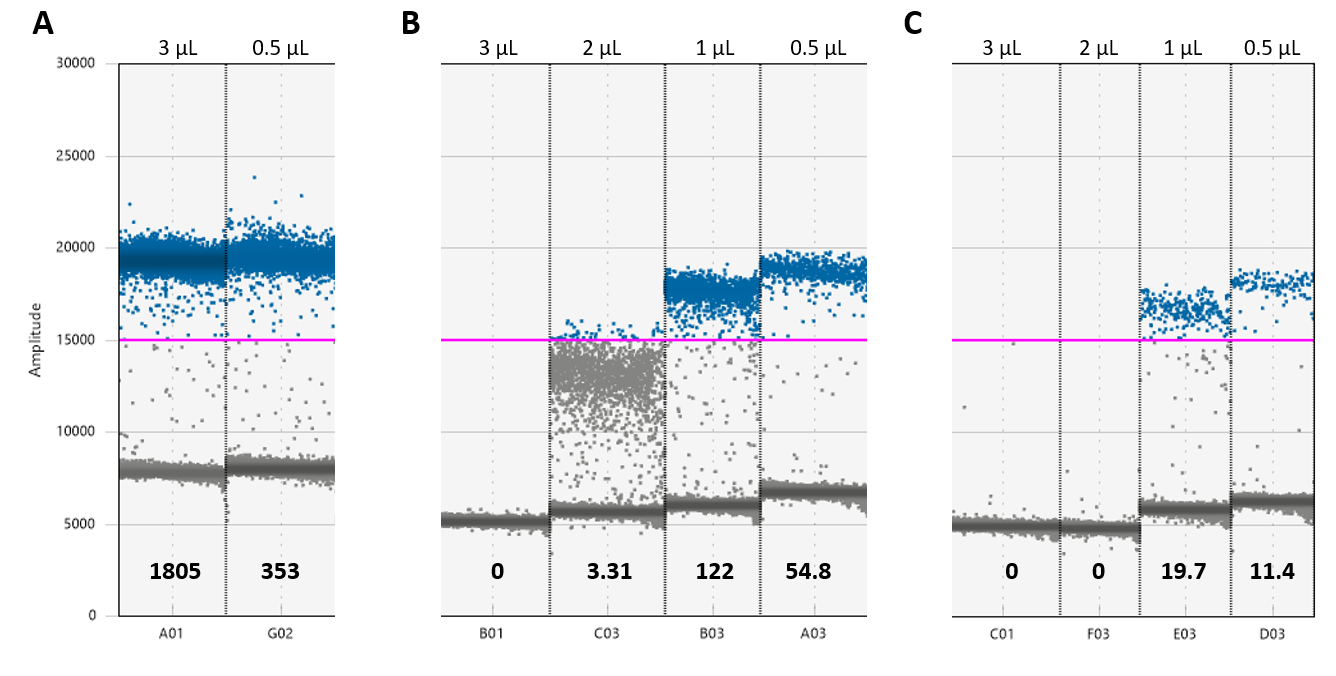
**

**Figure. S3** Optimization of *Sabella spallanzanii* digital droplet polymerase chain reaction (direct-ddPCR) assay: comparing different volumes of the sample added to the direct-ddPCR to detect free-floating extra-cellar environmental DNA (free-eDNA) from three different matrixes; sterile water (MilliQ) (**A**), artificial seawater (31 ppt) (**B**), and artificial seawater (34 ppt) (**C**). The sample volume added to the reaction well is shown at the top of each well in the Figure, either 3, 2, 1 or 0.5 µL. The estimated copies/µL for each replicate are shown near the bottom of each Figure, as determined by the QuantaSoft^TM^ Analysis Pro software (Version 10.596.0525). The y-axis shows units of amplitude for the fluorescent signal. The estimated copies/µL of DNA are measured by the number of target-positive droplets (shown in blue) above the baseline of target-negative droplets (shown as the dark layer), as visualized and analyzed using the software. The purple line shows the upper threshold of the target-negative droplets calculated from the negative controls, the threshold set to 15000 based on negative and positive controls, not seen here.


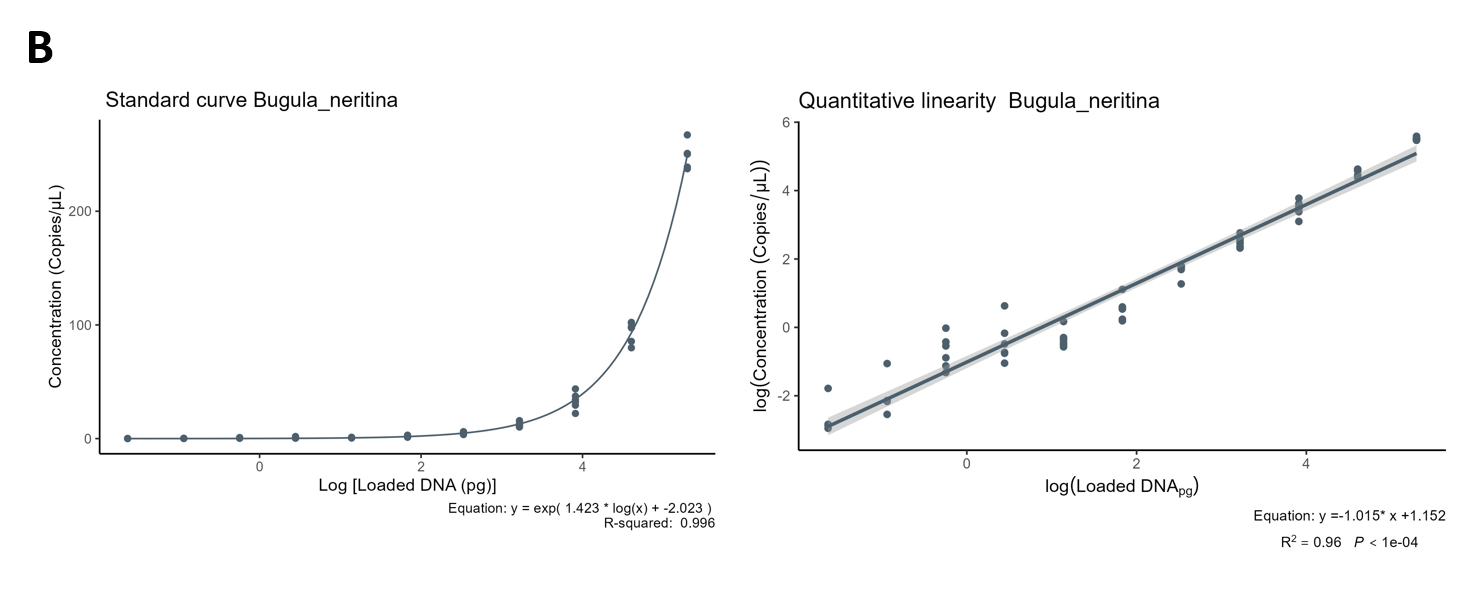

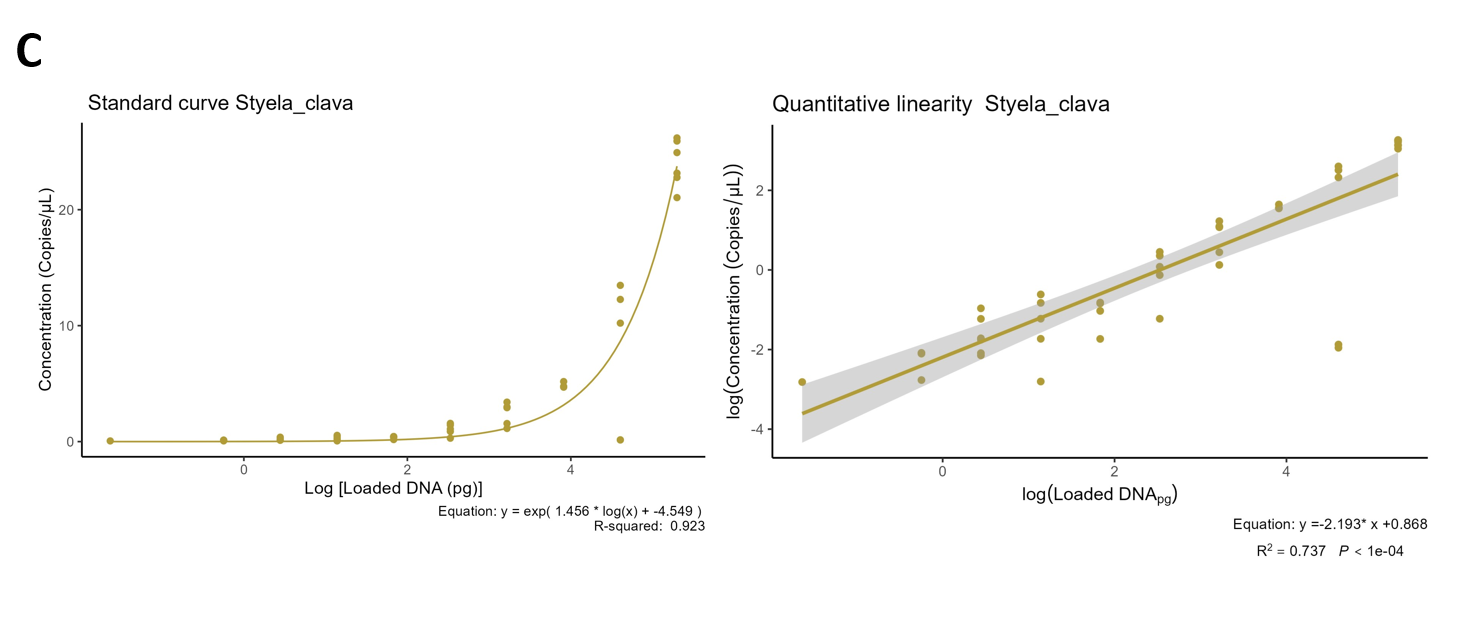

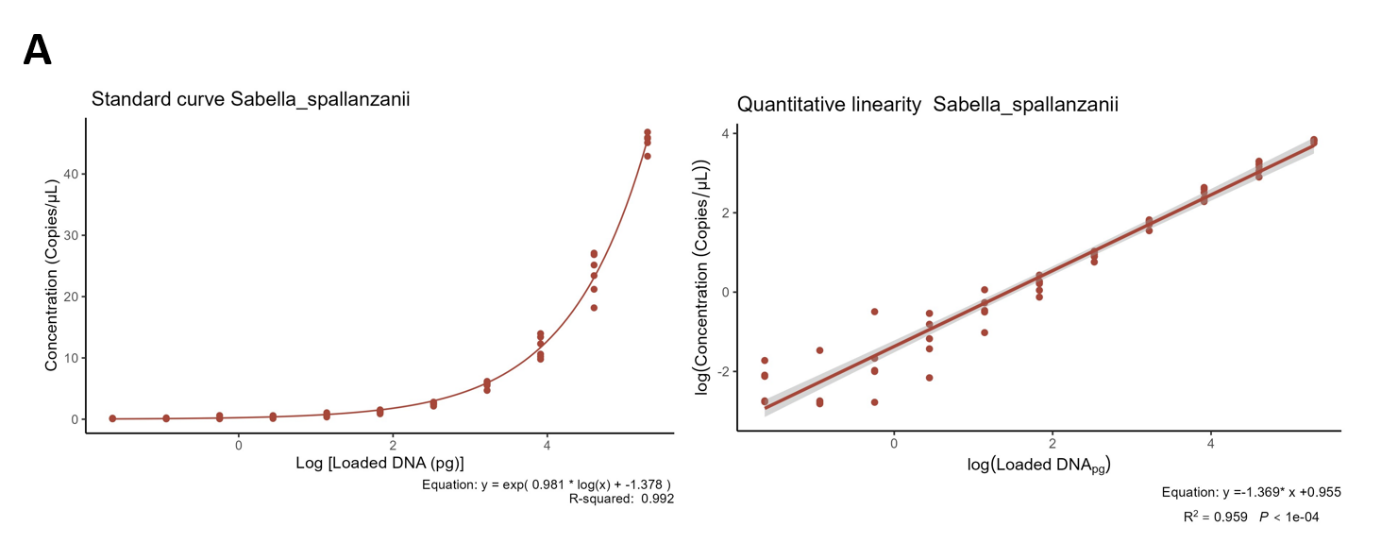


**Figure S4**. Visualization of the optimized *Sabella spallanzanii* **(A)***, Bugula neritina* **(B)***,* and *Styela clava* **(C)**. *Cytochrome c Oxidase* subunit 1 (COI) gene direct digital droplet polymerase chain reaction (direct-ddPCR) standard curve and reaction efficiency as described by quantitative linearity. The standard was 2x dilution starting with ~200pg of genomic DNA isolated from tissue samples. The standard curves were fitted using an exponential model with a concentration of COI (copies/µL) measured by direct-ddPCR on the y-axis against the log-transformed value of the loaded DNA (ng) of the standards on the x-axis. Both the equation of the line and goodness of fit (R-squared value (R^2^)) are shown on the plot. The quantitative linearity was assessed by plotting the log10-transformed COI copy concentration measured by direct-ddPCR plotted against the corresponding log10-transformed inputted ng of DNA and fitted with linear regression. The equation of the linear, the goodness of fit (R^2^) and the associated p-value are included in the plot. Note different y and x-axis scales.


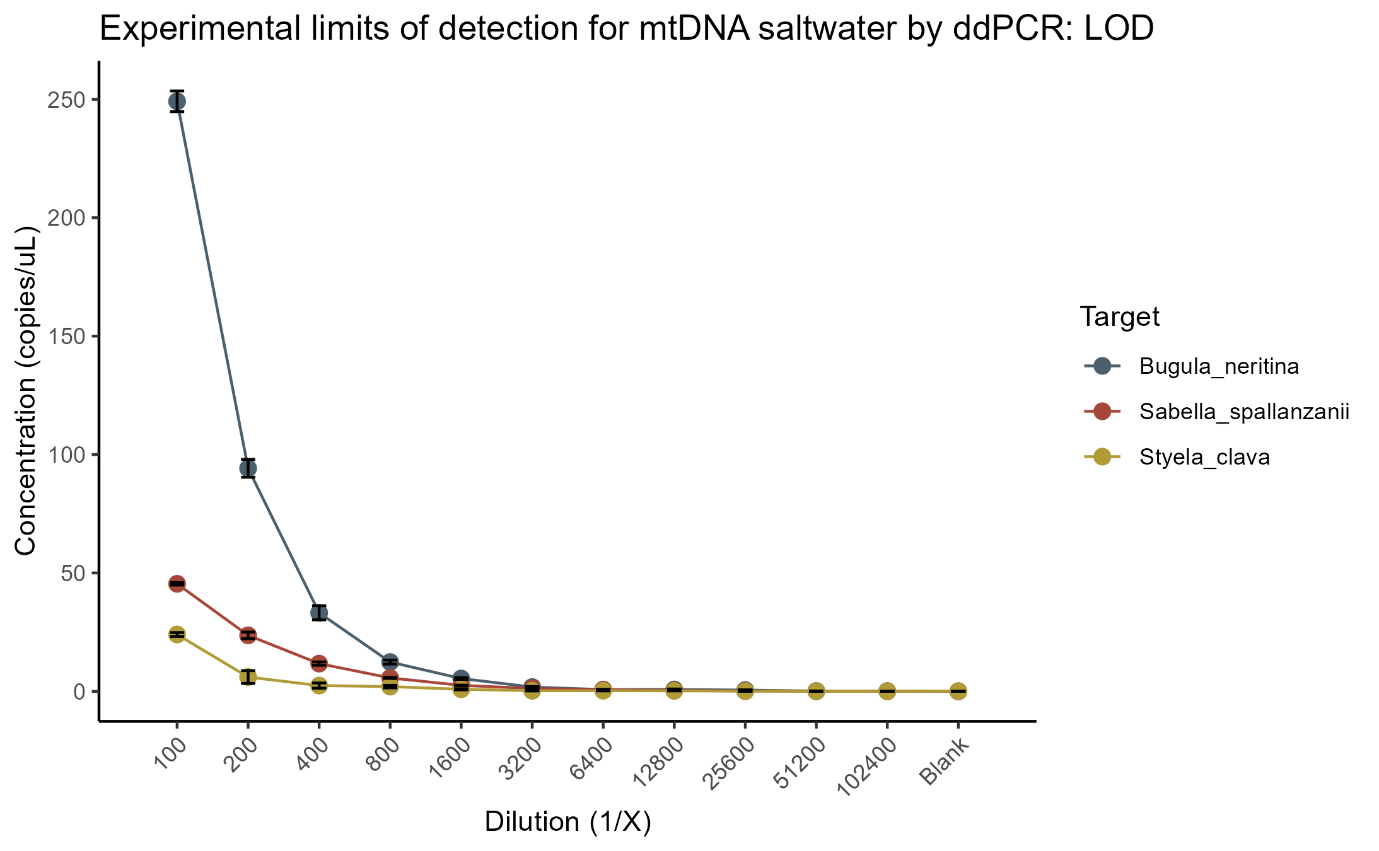


**Figure S5.** Experimental limit of detection (LOD) for the *Sabella spallanzanii*, *Styela clava* and *Bugula neritina* *Cytochrome c Oxidase* subunit 1 (COI) gene by direct digital droplet polymerase chain reaction (direct-ddPCR) using a 2x dilution starting with ~200pg of genomic DNA isolated from tissue samples. The direct-ddPCR was run with six replicates using either a duplex probe reaction mix or a singleplex EvaGreen reaction mix to amplify species-specific fragments from the COI gene. Results are grouped by target species. Vertical lines represent standard errors.


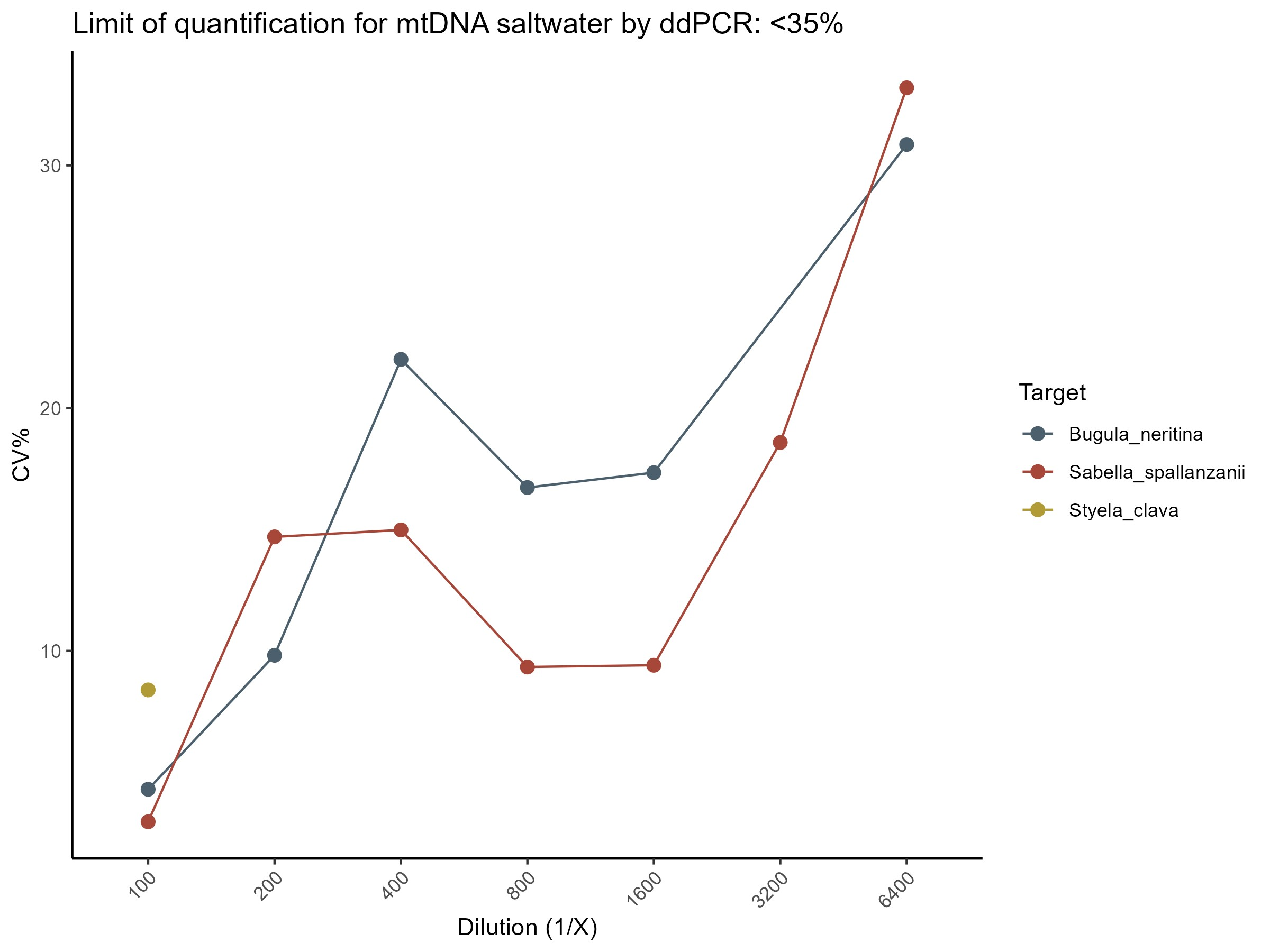


**Figure S6.** Experimental limit of quantification (LOQ) for the *Sabella spallanzanii*, *Styela clava* and *Bugula neritina* *Cytochrome c Oxidase* subunit 1 (COI) gene by direct digital droplet polymerase chain reaction (direct-ddPCR) using a 2x dilution starting with ~200pg of genomic DNA isolated from tissue samples. The y-axis is the average coefficient of variation (CV) percentage. The LOQ, in this manuscript, is defined as the lowest standard concentration that could be quantified with a CV value below 35%; therefore, only dilutions with CV<35% were included in the plot. The direct-ddPCR was run with six replicates using either a duplex probe reaction mix or a single plex EvaGreen reaction mix to amplify species-specific fragments from the COI gene.

**Table S1**

Average copies/µL of the *Cytochrome c Oxidase* subunit 1 (COI) gene present in the aquarium tanks for results for, Styela clava, *Bugula neritina* and Sabella spallanzanii. Results are based on average across all time points with the organism present and tanks. The direct digital droplet polymerase chain reaction (direct-ddPCR) detection limit is defined as 0.130 copies /µL for S. spallanzanii and 0.08 copies/µL for B. neritina and S. clava in this study.

| Species | Average copies/µL | STDEV | Number of positive detections | Detection Percentage (%) |
| --- | --- | --- | --- | --- |
| *Styela clava* | 0.377 | 0.254 | 9 | 6.25 |
| *Bugula neritina* | 0.224 | 0.136 | 35 | 24.2 |
| *Sabella spallanzanii* | 2.17 | 3.04 | 107 | 74.3 |

Table notes: Detection percentage was calculated by dividing the number of positive detections over the total number of samples 144 (8-time points x 3 tanks x 6 replicates)

**Table S2**

Average copies of the Cytochrome c Oxidase subunit 1 (COI) gene present tanks with high, medium, and low biomass for Sabella spallanzanii, *Bugula neritina and* Styela clava. Results are based on average across all points with the organism present and tanks. The direct digital droplet polymerase chain reaction (direct-ddPCR) detection limit is defined as 0.130 copies/µL for S. spallanzanii and 0.08 copies/µL for B. neritina and S. clava in this study.

| **Species** | **Tank Biomass** | **Average copies/µL** | **Maximum copies/µL** | **n** | **Detection Percentage (%)** |
| --- | --- | --- | --- | --- | --- |
| Sabella spallanzanii | high | 3.15 | 13.3 | 39 | 81.3 |
|  | medium | 2.79 | 8.78 | 32 | 66.7 |
|  | low | 0.545 | 1.31 | 36 | 75.0 |
| *Bugula neritina* | high | 0.253 | 0.532 | 19 | 39.6 |
|  | medium | 0.203 | 0.711 | 13 | 27.1 |
|  | low | 0.131 | 0.140 | 3 | 6.25 |
| *Styela clava* | high | 0.518 | 0.918 | 2 | 4.17 |
|  | medium | 0.117 | 0.117 | 1 | 2.08 |
|  | low | 0.374 | 0.599 | 6 | 12.5 |

Table notes: Detection percentage was calculated by dividing the number of positive detections over the total number of samples 48 (8-time points x 6 replicates)

**Table S3**

Exponential modelling results depicting the persistence of free-floating extra-cellar environmental DNA (free-eDNA) *Cytochrome c Oxidase* subunit 1 (COI) gene for *Sabella spallanzanii*, *Bugula neritina*, and *Styela clava* once organisms were removed from the tanks. These values were estimated by fitting the exponential model, y=ae^bx^, to the raw data. The direct digital droplet polymerase chain reaction (direct-ddPCR) detection limit is defined as 0.130 copies/µL for *S. spallanzanii* and 0.08 copies/µL for *B. neritina* and *S. clava* in this study. Calculations are based on data after organismal removal and are averaged across all tanks (biomass).

| Species | Coefficient a | Coefficient b | r-squared | Akaike information criterion (AIC) | Bayesian information criteria (BIC) |
| --- | --- | --- | --- | --- | --- |
| *Styela clava* | 0.332 | -0.0614 | 0.823 | -9.17 | -11.0 |
| Sabella spallanzanii | 7.44 | -0.0340 | 0.974 | 12.8 | 12.1 |
| *Bugula neritina* | 0.784 | -0.0411 | 0.855 | -4.26 | -4.89 |


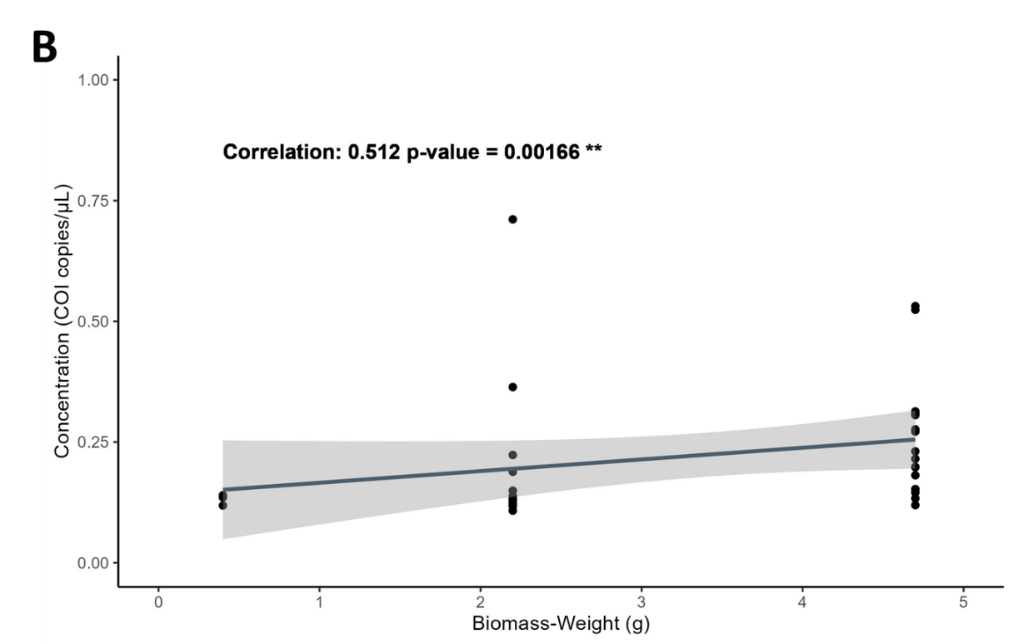

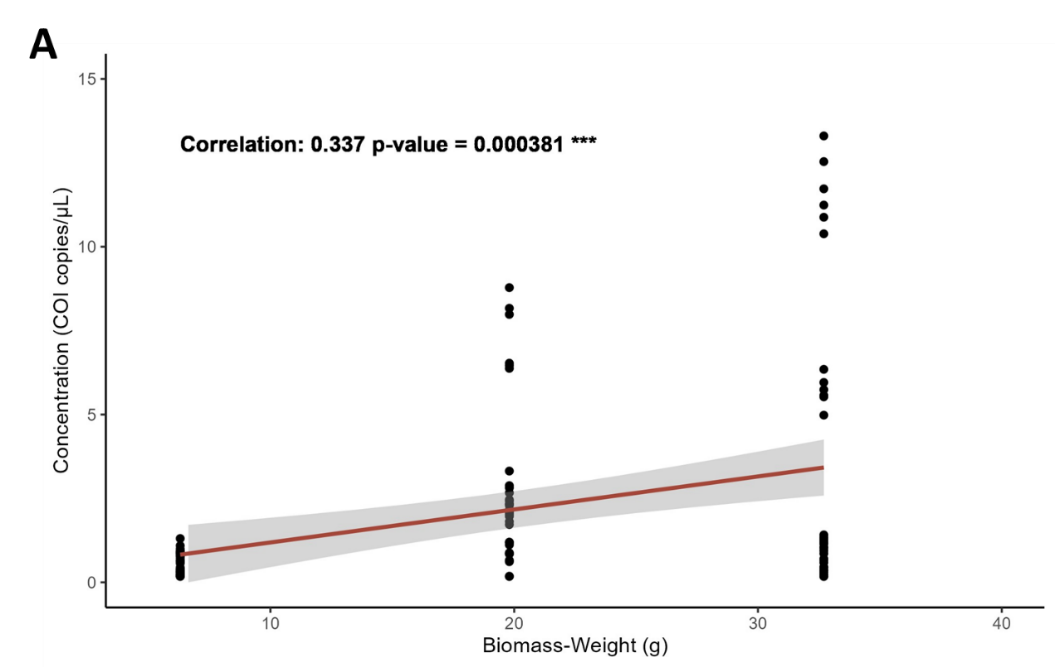


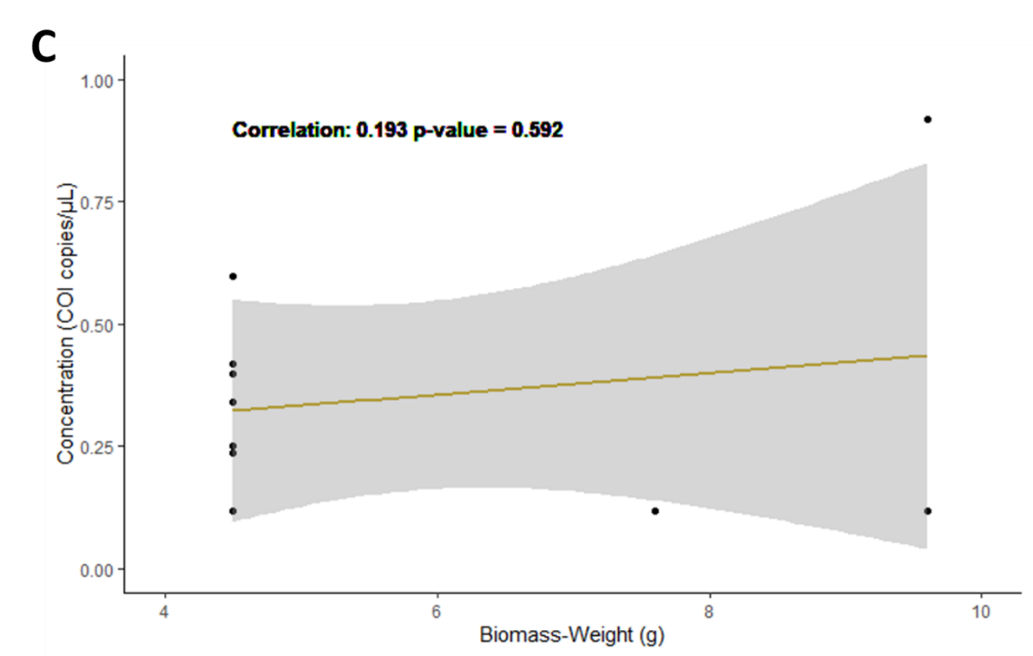


**Figure S7**. Visualization of the correlation between biomass, the weight of all species in the tank, and the concentration (copies/µL) of the *Cytochrome c oxidase* subunit 1 (COI) present in the aquarium for *Sabella spallanzanii* **(A)***, Bugula neritina* **(B)***,* and *Styela clava* **(C)**. The correlation value and significance (p-value), determined using R version 4.2.1, are included in each plot in the upper left corner. Significance levels are denoted by asterisks (* = p-value < 0.05, ** = p-value < 0.01, and *** = p-value < 0.001). The plots show data for time points when organisms were present in the aquarium. Note different y and x-axis scales.

**Table S4**

Results of the mixed-model analysis and fit statistics examining the effects of weight, tank, and species (*Sabella spallanzanii, Bugula neritina,* and *Styela clava)* on free-floating extra-cellar environmental DNA (free-eDNA) *Cytochrome c Oxidase* subunit 1 (COI) (copies/µL) calculated by direct digital droplet polymerase chain reaction (direct-ddPCR)

| Summary data for generalized linear mixed model (Formula: inv_Concentration ~ Weight + (1 \| Tank) + (1 \| Target Species) | | | | | | |
| --- | --- | --- | --- | --- | --- | --- |
| Factor | Effect | Estimate | Variance | Standard Error | t-value | p-values |
| Intercept | Fixed | 5.001 | NA | 1.309 | 3.82 | 00000133*** |
| Weight | Fixed | -0.066 | NA | 0.063 | -1.057 | 0.290 |
| Tank | Random | NA | 0.442 | 0.6651 | NA | NA |
| Target Species | Random | NA | 1.08 | 1.04 | NA | NA |
| Residual variance | Random | NA | 0.494 | 0.703 | NA | NA |
| Mixed Model fit statistics | | | | | | |
| AIC:189.42 | | | | | | |
| BIC:198.11 | | | | | | |

Note: To fit a gamma distribution and avoid a singular model, the concentration was transformed by taking the reciprocal of the concentration (1/Concentration (COI copies/µL). The number of observations is 42, and there are 3 groups for both Tank and Target Species. Significance levels are indicated by asterisks (* = p-value < 0.05, ** = p-value < 0.01, and *** = p-value < 0.001). Notably, the model incorporating the random effects of Tank and Target Species outperformed the model without these effects, as evidenced by the ANOVA test (p = 0.023*).
