# Supplementary material for: Drop it all: extraction-free detection of targeted marine species through optimized direct droplet digital PCR
Source: PeerJ. 2024 Feb 23;12:e16969. doi: 10.7717/peerj.16969 (PMC10896080; doi:10.7717/peerj.16969)
Supplement: Supplemental Information 4 [file peerj-12-16969-s004.docx]

**Supplemental Information for:**

Drop it all: Extraction-free detection of targeted marine species through optimized direct-digital droplet PCR

**Supplementary Information-Supplementary File 2**

In-vitro optimization trials for free-floating environmental DNA (free-eDNA) detection in seawater: **Summary table**

**Table S1**

Summary table of the methods for testing and optimizing detection of free-floating extra-cellar environmental DNA (free-eDNA) in seawater using direct digital droplet polymerase chain reaction (direct-ddPCR). The table describes the reagents or conditions used during optimization and the targeted species tested. Other details are also listed, including the water matrixes used during testing, the sample volume, the reaction annealing temperature, and the overall outcome. Any natural seawater used as a water matrix was collected from the local harbour (Nelson Marina, Nelson, New Zealand (41.26° S, 173.28° E)).

| Factor tested to optimize direct-ddPCR reaction | Water matrix used | Reagent used and concentration added to the direct-ddPCR reaction^†^ | Volume of sample added to direct-ddPCR assay (µL) | Anneal Temperature (°C) | Species used | Outcome |
| --- | --- | --- | --- | --- | --- | --- |
| PCR additives | artificial seawater (36.1) | BSA (0.2 mg/mL) + 0.75% Tween-20 + 0.8% Glycerol | 3 | 60 | *Sabella spallanzanii* | No detection |
|  |  | Tween-20: 0.75% | 3 | 60 | *Sabella spallanzanii* | No detection |
|  |  | DMSO (Dimethylsulfoxide) 5% | 3 | 60 | *Sabella spallanzanii* | No detection |
|  |  | Glycerol 8% | 3 | 60 | *Sabella spallanzanii* | No detection |
| ddPCR Mastermix | Sterile (MiliQ), Tap Water & Artificial Seawater (36.1, 31.9 & 39.8 ppt) | ddPCR Supermix for Probes (No dUTP): 1X | 3 | 60 | *Sabella spallanzanii* | No detection in seawater |
|  |  | ddPCR Multiplex Supermix (No dUTP): 1X | 6 | 60 | *Sabella spallanzanii* | No detection in seawater |
|  |  | ddPCR Supermix residual DNA quantification: 1X | 3 | 60 | *Sabella spallanzanii* | No detection in seawater |
|  |  | ddPCR Multiplex Supermix (No dUTP): 1X | 3 | 57 | *Sabella spallanzanii & Styela clava* | Detection in all water matrixes |
| Buffer-Added directly to the ddPCR | Artificial seawater (31.1 ppt, 34.0 ppt) | Trizma Base Buffer (40mM pH 8.0) | 3 | 60 | *Sabella spallanzanii* | No detection |
|  |  | 0.5X TAE buffer | 3 | 60 | *Sabella spallanzanii* | No detection |
|  |  | 1M HEPES Buffer (40mM, pH 7.5) | 3 | 60 | *Sabella spallanzanii* | No detection |
| Buffering water solution, diluting prior to collecting aliquot | Artificial seawater (31.1 ppt, 34.0 ppt) | Trizma Base Buffer (40mM pH 8.0) | 3 | 60 | *Sabella spallanzanii* | Detection |
|  |  | 0.5X TAE buffer | 3 | 60 | *Sabella spallanzanii* | Detection |
|  |  | 1M HEPES Buffer (40mM, pH 7.5) | 3 | 60 | *Sabella spallanzanii* | Detection |
| pH adjustment | Artificial seawater (31.1 ppt, 34.0 ppt) | 1 KOH: 4µL | 3 | 60 | *Sabella spallanzanii* | Negatively affected assay, no detection |
|  |  | 1 KOH: 3µL | 3 | 60 | *Sabella spallanzanii* | Negatively affected assay, no detection |
|  |  | 1 KOH: 2µL | 3 | 60 | *Sabella spallanzanii* | Negatively affected assay, no detection |
|  |  | 1 KOH: 1µL | 3 | 60 | *Sabella spallanzanii* | Some detection, most also impacted reaction |
| Sample volume | Sterile (MiliQ) | NA | 3 | 60 | *Sabella spallanzanii* | Detection |
|  |  |  | 0.5 | 60 | *Sabella spallanzanii* | Detection |
|  | Artificial seawater (31.1 ppt, 34.0 ppt) | NA | 3 | 60 | *Sabella spallanzanii* | No Detection |
|  |  |  | 2 | 60 | *Sabella spallanzanii* | Some detection in 31.1 ppt |
|  |  |  | 1 | 60 | *Sabella spallanzanii* | Detection |
|  |  |  | 0.5 | 60 | *Sabella spallanzanii* | Detection |
| Annealing temperature | Sterile (MiliQ), Tap Water & Artificial Seawater (31.1 & 34.0 ppt), Natural Seawater | NA | 1 | temperature gradient: 64.4-55 | *Sabella spallanzanii, Styela clava* | Detection at all temperatures |
|  | Natural Seawater |  |  |  | *Bugula neritina* | Detection at all temperatures |
| Probe and Primer Concentrations | Sterile (MiliQ) & Natural Seawater | 900 nM primer + 250 nM probe | 1 | 57 | *Sabella spallanzanii & Styela clava* | Detection |
|  |  | 476 nM primer + 250 nM probe | 1 | 57 |  | Detection |
|  |  | 476 nM primer + 476 nM probe | 1 | 57 |  | Detection |
|  | Natural Seawater | 476 nM primer ^‡^ | 1 | 57 | *Bugula neritina* | Detection |
|  |  | 250 nM primer ^‡^ | 1 | 57 |  | Detection |

**Table Notes:** ^†^Primer and probe concentration based on final concentration in the direct-ddPCR reaction. ^‡^For *Bugula neritina,* the assay is an EvaGreen ddPCR assay; therefore, there is no probe.

In-vitro optimization trials for free-floating extra-cellar environmental DNA (free-eDNA) detection in seawater: in-depth methods and results

There was no amplification in samples collected from the negative control or the no-template (NTC) direct-digital droplet polymerase chain (ddPCR) controls throughout the optimization trial. Based on our experience and observation of ddPCR noise (e.g., proportions of fluorescing droplets in water blanks), the detection for all assays was set above the maximum value of the negative controls in the experiment. Of note, unless stated, was able to consistently detect species-specific free-eDNA in purified water (Milli-Q®; Millipore Sigma™, USA) water samples, which were always added as a positive control.

1. Pilot One: Testing if free-eDNA can be detected in purified water.
   1. **Method:** Three Nalgene™ square polycarbonate bottles (Thermo Fisher Scientific, USA) were filled: one with 250 mL purified water (Milli-Q®; Millipore Sigma™, USA) as a negative control, another with the same water plus *Sabella spallanzanii* (Gmelin, 1791) - a large Mediterranean fan worm, preserved in 99% ethanol (collected from Golden Bay 11.4.17), and a third with water spiked with 500 ng of *S. spallanzanii* genomic DNA. To simulate eDNA release, organisms were shaken within the bottles for several minutes. Subsequently, 20 µL water aliquots were collected from each bottle and added to the optimized *S. spallanzanii*-specific ddPCR reaction following Wood et al. 2020. The direct-ddPCR reaction included 10 μL of 2X ddPCR Supermix for Probes (No dUTP) (BioRad, USA), 1 μL of each primer and probe at 10 pmol, 3 μL of the collected water sample, and sterile water for a total volume of 21 μL. The assay used a cycle of 95 °C for 10 min, 40 cycles of 95 °C for 30 s, 60 °C for 1 min, and a final enzyme deactivation step at 98 °C for 10 min. Each direct-ddPCR plate run included at least one negative control (RNA/DNA-free water Life Technologies) and two positive controls (genomic DNA extracted from *S. spallanzanii* and environmental DNA extracted using conventional extraction methods).
   2. **Results:** Positive *S. spallanzanii* free-eDNA detection in the bottle spiked with *S. spallanzanii* organism, and from the bottle spiked with extracted genomic *S. spallanzanii* DNA. No detection in the negative controls.
2. Pilot two: Testing if free-eDNA can be detected in local seawater.
   1. **Method:** Four individual Nalgene™ square polycarbonate bottles: one with 250 mL purified water as a negative control, another with the water plus a *Sabella spallanzanii* organism, a third with locally collected seawater and a fourth with the same water plus a *Sabella spallanzanii* organism (collected from Golden Bay on 11.4.17). To simulate eDNA release, the organisms were vigorously shaken within the bottles for several minutes. Subsequently, 20 µL water aliquots were collected from each bottle and directly added to the optimized *S. spallanzanii-s*pecific ddPCR reaction following Wood et al. 2020. The assay and conditions mirrored those in Pilot 1. Waters were run in duplicate, and samples from bottles with organisms were tested diluted 1:10 and undiluted. Each direct-ddPCR plate run included at least one negative control and two positive controls.
   2. **Results**: Positive *S. spallanzanii* free-eDNA detection in undiluted and diluted MiliQ samples, but no positive detection for *S. spallanzanii* eDNA in saltwater.
3. Troubleshooting Test One: Buffering water solutions, testing additives and comparing BioRad ddPCR master mix
   1. **Methods:** Tested different matrices using Nalgene™ bottles: tap water, purified water, and artificial seawater with varying salinities (Red Sea Salt-Copepod salt; Red Sea Germany). Salinity levels were adjusted to 31.9 ppt, 36.1 ppt, and 39.8 ppt. Optimization of the ddPCR reaction to remove inhibitors was explored at 36.1 ppt. This included the use of PCR additives such as glycerol (8%), dimethyl sulfoxide (DMSO) (5%), Tween-20 (1%), and combined additives: bovine serum albumin (BSA) (0.2 mg/mL) +1% Tween-20 + 8% Glycerol. Buffering solutions 1X Tris-acetate-EDTA (TAE pH 8.28) and 1M Tris aminomethane (Tris) pH 8.9 were tested. Three mastermix combinations, 4x ddPCR™ Multiplex Supermix (No dUTP), 2x ddPCR™ Supermix for Residual DNA Quantification and 2X ddPCR Supermix for Probes (No dUTP) from BioRad, were compared. Four Nalgene™ square polycarbonate bottles, each containing 250mL of one of the various water matrices mentioned earlier, were prepared. Negative control samples without the organism were collected. Subsequently, *S. spallanzanii* organisms were added to each Nalgene bottle (5 organisms total collected from Golden Bay 11.4.17, Westhaven Auckland 20.04.17, Opua 11.4.17 and Westhaven-unknown date). To simulate eDNA release, the organisms were vigorously shaken within the bottles for several minutes, and 20 µL aliquots were collected for ddPCR reactions. To buffer the water samples, 25 mL of 36.1 ppt water from the Nalgene bottle was transferred to a 50 mL falcon tube, and an additional 25 mL of buffer, either Trizmae Base Buffer (Tris) (1M pH 8.9) or TAE buffer (pH 8.28), was mixed in. Then, 20 µL aliquots were collected for ddPCR reactions. The assay cycling conditions mirrored those in Pilot 1 and 2. The direct-ddPCR reaction included 10 μL of 2X ddPCR mastermix (BioRad, USA), 1 μL of each primer and probe at 10 pmol, 3 μL of the collected water sample, and sterile water for a total volume of 21 μL. Except for the multiplex super mix, where 5 µL of ddPCR Multiplex super mix and 6 µL of the sample were added. The volume of the nuclease-free water was adjusted based on the type of additive used. For example, additives were added in the final concentrations of glycerol 0.8%, DMSO 0.5%, Tween 20 0.075%, and the combined additives BSA (0.2 mg/mL + 0.075% Tween20 + 0.08 Glycerol). Each combination of additive and buffering, along with negative and positive controls and samples without manipulation, were run with each of the ddPCR mastermixes, totaling at least 20 samples (20 conditions per mastermix). Each direct-ddPCR plate run included at least one negative control and two positive controls.
   2. **Results:** No positive detection with additives. Positive *S. spallanzanii* free-eDNA detection in MiliQ and tap water with the Residual and Supermix BioRad ddPCR master mix. Diluting 36.1 ppt samples with buffer resulted in detection when diluted with TAE buffer (pH 8.28) but not Tris (1M pH 8.9). BioRad Multiplex Supermix showed positive detection in MiliQ but not tap water. Although there was detection in the TAE-buffered samples, there was more noise and reduced amplitude when using the multiplex mix.
4. Troubleshooting Test two: Adjusting pH with bases and buffer solutions
   1. **Methods:** Tested different matrices using Nalgene™ bottles: purified water and artificial seawater with varying salinities. Salinity levels were adjusted to 31.1 ppt (pH 8.32) and 34.3 ppt (pH 8.36) based on the assessment of locally collected seawater. Optimization of the ddPCR reaction to remove inhibitors was explored at both salinities using 1M potassium hydroxide (KOH) to adjust the pH of the reaction. Buffering the solution was attempted with 1X TAE buffer (pH 8.28) and Tris (80 mM pH 8.17). Three Nalgene™ square polycarbonate bottles were prepared, each containing 250 mL of the various water matrices mentioned earlier. Negative control samples without the organism were collected. Subsequently, *S. spallanzanii* organisms were added to each Nalgene bottle (2 organisms total collected from Golden Bay 11.4.17 and Westhaven Auckland 20.04.17). To simulate eDNA release, the organisms were vigorously shaken within the bottles for several minutes, and 20 µL aliquots were collected for ddPCR reactions. To buffer the water samples, 25 mL of artificial saltwater solutions from the Nalgene bottle was transferred to a 50 mL falcon tube, and an additional 25 mL of buffer, either Tris (80 mM pH 8.17) or 1X TAE buffer (pH 8.28), was mixed in. Then, 20 µL aliquots were collected for ddPCR reactions. The assay cycling conditions mirrored those in pilot experiments. The direct-ddPCR reaction included 10 μL of 2X ddPCR Supermix for Probes, 1 μL of each primer and probe at 10 pmol, 3 μL of the collected water sample, and sterile water for a total volume of 21 μL. Adjustments were made for the amount of water based on the amount of KOH added, and additionally, we chose to add the buffer solution to the ddPCR mastermix instead of water (5 µL) for some of the samples. KOH was added increasingly from 1 µL to 4 µL. Each direct-ddPCR plate run included at least one negative control and two positive controls.
   2. **Results:** Buffering the water samples before adding directly to the ddPCR reaction resulted in positive *S. spallanzanii* free-eDNA detection in the 31.1 and 34.3 ppt artificial seawater solutions. However, the buffer solution did not yield a positive detection when added directly to the ddPCR mastermix. Additionally, although 1 µL of KOH did lead to *S. spallanzanii* free-eDNA detection in the 31.1 ppt seawater solution, there was no detection in the 34.3 ppt solution, and volumes higher than 1 µL had a negative impact on the ddPCR reaction.
5. Troubleshooting Test two: Adjusting pH with buffers and adjusting sample volume
   1. **Methods:** Tested different matrices using Nalgene™ bottles: purified water and artificial seawater with varying salinities. Salinity levels were adjusted to 31.1 ppt (pH 8.32) and 34.3 ppt (pH 8.36) based on the assessment of locally collected seawater. Optimization of the ddPCR reaction to remove inhibitors was explored by adjusting the sample volume input and buffering the solutions. In this final troubleshooting experiment, various sample volumes and buffering solutions, including 1X TAE buffer (pH 8.41), Tris (80 mM pH 8.17), and 1M 4-(2-hydroxyethyl)-1-piperazineethanesulfonic acid (HEPES) pH 7.51, were tested to determine their impact on reaction performance. Three Nalgene™ square polycarbonate bottles were prepared, each containing 250 mL of one of the various water matrices mentioned earlier. Negative control samples without the organism were collected. Subsequently, *S. spallanzanii* organisms were added to each Nalgene bottle (3 organisms total collected from Golden Bay 11.4.17). To simulate eDNA release, the organisms were vigorously shaken within the bottles for several minutes, and 20 µL aliquots were collected for ddPCR reactions. To buffer the water samples, 25 mL of artificial saltwater solutions from the Nalgene bottle was transferred to a 50 mL falcon tube, and an additional 25 mL of buffer, either Tris (80 mM pH 8.0), 1X TAE buffer (pH 8.28), or HEPES (1M pH 7.51), was mixed in. Then, 20 µL aliquots were collected for ddPCR reactions. The assay cycling conditions mirrored those in pilot experiments. The direct-ddPCR reaction included 10 μL of 2X ddPCR Supermix for Probes (No dUTP) (BioRad, USA), 1 μL of each primer and probe at 10 pmol, 0.5-3 µL of the collected water sample, and sterile water for a total volume of 21 μL. Adjustments were made for the amount of water based on the input sample (ranging from 0.5-3). Additionally, to buffering solutions prior to aliquoting samples, some samples had the buffer solution was added to the ddPCR mastermix instead of water (5 µL). Each direct-ddPCR plate run included at least one negative control and two positive controls.
   2. **Results:** Buffering the water samples before adding them directly to the ddPCR reaction resulted in positive *S. spallanzanii* free-eDNA detection in the 31.1 and 34.3 ppt artificial seawater solutions. However, similar to the previous experiment, there was no detection when the buffer solution was added directly to the ddPCR reactions. The results suggested that a lesser volume removed some of the inhibitory properties of the artificial seawater samples, with positive detection when adding only 0.5 and 1 µL of sample for the 34.3 ppt, and some positive detection when adding 0.5, 1, and 2 µL of sample for the 31.1 ppt solution. Buffering the solution with TAE and HEPES before aliquoted (loaded 3 µL of diluted solution), compared to loading only 1 µL of undiluted sample, yielding similar *S. spallanzanii* free-eDNA results for 31.1 ppt and 34.0 ppt, respectively. Adding the buffer instead of water directly to the ddPCR had no benefit.
6. **Finalizing Reaction:** Optimization steps, including temperature gradient, primer-probe mix, and testing with natural seawater
   1. **Method:** In these final experiments, we repeated the troubleshooting experiment in locally collected seawater and completed the final optimization to enhance the assay. Different matrices were tested, including purified water, locally collected seawater, and artificial seawater with varying salinities. Salinity levels were adjusted to 31.1 ppt (pH 8.33) and 34.8 ppt (pH 8.37) based on the assessment of locally collected seawater. Four Nalgene™ square polycarbonate bottles were prepared, each containing 250 mL of one of the various water matrices mentioned earlier. Negative control samples without the organism were collected. Subsequently, *S. spallanzanii* organisms were added to each Nalgene bottle (4 organisms total collected from Golden Bay 11.4.17 and Sabella Westhaven Auckland 20.07.17). To simulate eDNA release, the organisms were vigorously shaken within the bottles for several minutes, and 20 µL aliquots were collected for ddPCR reactions. The assay conditions were set up as a temperature gradient: hold at 95°C for 10 min, 40 cycles of 94°C for 30 s, 65-55°C (65, 64.4, 63.1, 59, 57.1, 55.8, 55) temperature gradient for 1 min, and a final enzyme deactivation step at 98°C for 10 min. The direct-ddPCR reaction included 10 μL of 2X ddPCR Supermix for Probes (No dUTP) (BioRad, USA), 1 μL of each primer and probe at 10 pmol, 1 µL of the collected water sample, and sterile water for a total volume of 21 μL, based on the previous experiment. Each direct-ddPCR plate run included at least one negative control and one positive control; in addition, 3µL of sample seawater was added instead of 1 µL as an additional check.
   2. **Results:** Consistently obtained detection from purified water, 31, 34 ppt, and seawater samples when adding 1 µL *S. spallanzanii* free-eDNA at all temperature gradients around similar copies/µL. Based on the results from the mean amplitude total and the concentrations across all four water matrices, 57°C was chosen as the optimized temperature for the detection of *S. spallanzanii* free-DNA.
   3. **Alternations for Other Species:** This experiment was repeated for two other marine non-indigenous species (NIS) species, *Styela clava* (Herdman, 1881) - a leathery club tunicate, and *Bugula neritina* (Linnaeus, 1758) - a bush-like, calcified bryozoan. In these cases, these species were also preserved in ethanol and placed in various Nalgene bottles with seawater to try and get species-specific free eDNA detection (*Styela Clava* collected from Nelson Harbour 30Mar22 and *Bugula Neritina* collected from the Nelson Harbour MKS 04Apr22). The same temperature gradient was used, testing volume concentrations between 0.5 - 3 µL, resulting in the same results as *S. spallanzanii*. Note that the ddPCR reactions for *S. clava* and *B. neritina* were based on the results above and the optimized assay developed by Gillum et al. 2014 and Kim et al. 2018.
   4. **Additional Tests:**
      1. Tested 4x ddPCR™ Multiplex Supermix (No dUTP) for *S. spallanzanii* and *S. clava* - but instead added less sample volume and found that this mastemix worked with up to 3 µL of the sample.
      2. Tested various primer-probe concentrations for the *S. spallanzanii* and *S. clava* ddPCR assays (900 nM primer + 250 nM probe, 476 nM primer + 250 nM probe, 476 nM primer + 476 nM probe). The best amplitude change was still the original probe and primer concentration (476 nM primer + 476 nM probe) with 1 µL of the sample. Even with changing primer and probe concentration and using the optimized temperature of 57 °C, using 3 µL yielded no free-eDNA detection in the natural seawater.
      3. *Bugula neritina* optimized reaction is an EvaGreen assay, so we also tested this assay using the experiment described above. Using the following cycle conditions: hold at 95 °C for 10 min, 40 cycles of 95 °C for 30 s, 57 °C 1 min, and a final signal stabilization and enzyme deactivation steps at 4 °C for 5 min and 90°C for 5 min. Also, we performed the same temperature gradient for this assay and, similar to *S. spallanzanii* and *S. clava* and, found that 57°C was optimal in terms of detected copies/µL and amplitude total. For the optimized *B. neritina* reaction, each direct-ddPCR reaction included 10 μL of 2X QX200™ ddPCR™ EvaGreen Supermix (BioRad, USA), 0.5 μL of each primer at 10 pmol, 1 μL of the collected water sample, and 9 μL of sterile water for a total volume of 21 μL. We also tried that assay with 1 µL of primer at 10 pmol but found that using only 250 nM instead of 475 nM slightly improved the background ddPCR noise and copies/µL of *B. neritina* free-eDNA.

In the final in-vitro experiment, all species were combined in one Nalgene bottle with natural seawater, and the assay was executed using a duplex assay for *S. spallanzanii* and *S. clava*, as well as a singleplex assay for *Bugula neritina*, incorporating all the optimized steps discussed earlier. Notably, consistent detection was achieved for all three species, and no interference was observed when placed in a shared Nalgene bottle. Notably, for S. spallanzanii and S. clava, no difference was observed between the singleplex and duplex reactions in terms of free-eDNA detection.
